# Panniculitis in a patient presenting with a pancreatic tumour and polyarthritis: a case report

**DOI:** 10.4076/1752-1947-3-7331

**Published:** 2009-07-06

**Authors:** Carolyn Chee

**Affiliations:** 1 Nottingham City Hospital, Nottingham University Hospitals NHS Trust City Campus, Hucknall Road, Nottingham NG5 1PB, UK

## Abstract

**Introduction:**

Panniculitis is a rare manifestation of pancreatic disease. Rarer still is the association of panniculitis, pancreatitic disease and polyarthritis. A literature search revealed less than five cases of pancreatic panniculitis associated with pancreatic tumour and polyarthritis.

**Case presentation:**

An 84-year-old Caucasian man presented with epigastric pain, weight loss, polyarthritis and multiple discharging nodules. A computed tomography scan revealed a mass in the head of the pancreas. Histology of the cutaneous lesions confirmed the diagnosis of pancreatic panniculitis.

**Conclusion:**

Pancreatic panniculitis can clinically present in many ways to clinicians across a broad scope of specialties. Knowledge and understanding of the association between panniculitis and polyarthritis with pancreatic disease may aid rapid diagnosis and management.

## Introduction

Pancreatic panniculitis is a rare disease involving fat necrosis in the panniculus in association with pancreatic disease. The rarity of this condition is best described by Mullin et al. who reported one case of panniculitis out of 893 patients with various pancreatic diseases [1]. We report a man presenting with panniculitis, pancreatic tumour and polyarthritis with a fatal outcome. Awareness of the rare skin manifestation observed and the potentially fatal consequences, can guide physicians to rapid diagnosis and management.

## Case presentation

An 84-year-old Caucasian man presented with a 4-week history of severe abdominal pain radiating to his back and with haematuria. He was receiving long-term warfarin treatment for atrial flutter and his international normalised ratio on admission was 17.6. He complained of nausea and weight loss, but reported no recent change in bowel habit.

On examination, the patient was cachectic and pyrexial. He had epigastric and right upper quadrant tenderness. Of note was swelling of his right carpometacarpal, bilateral thumb and index finger metacarpal joints.

**Figure 1:**
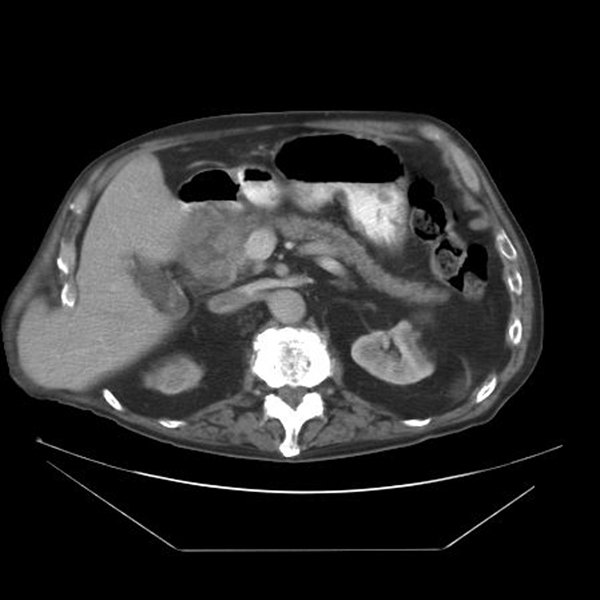
**Computed tomography scan of the abdomen showing a mixed attenuation mass of the head of pancreas**.

Laboratory investigations revealed a white-cell count of 12.6 × 10^9^/L, C-reactive protein of 198 mg/L, gamma-glutamyl transpeptidase of 273, raised serum lipase of 17362 U/L and serum amylase of 619 U/L. Chest and abdominal X-rays were normal. The abdominal computed tomography (CT) scan showed a 9 × 8 cm mixed attenuation mass initially thought to represent pancreatic head haematoma (Figure 1). The patient's warfarin treatment was discontinued and he was managed conservatively with intravenous fluids, analgesia and bed rest. Levels of the tumour markers carcinoembryonic antigen (CEA) and CA19-9 were normal. A follow-up scan 8 weeks later revealed that the mass had not resolved and was now more likely to represent a tumour of the head of the pancreas or a pancreatic pseudocyst. The patient declined an endoscopic ultrasound.

**Figure 2:**
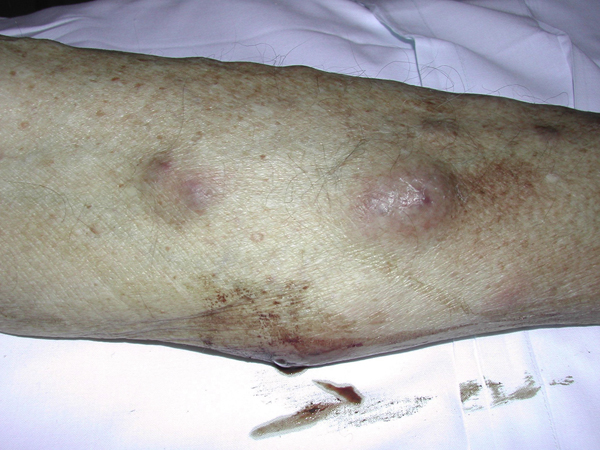
**Multiple reddish-purple nodules on lower limb**.

**Figure 3:**
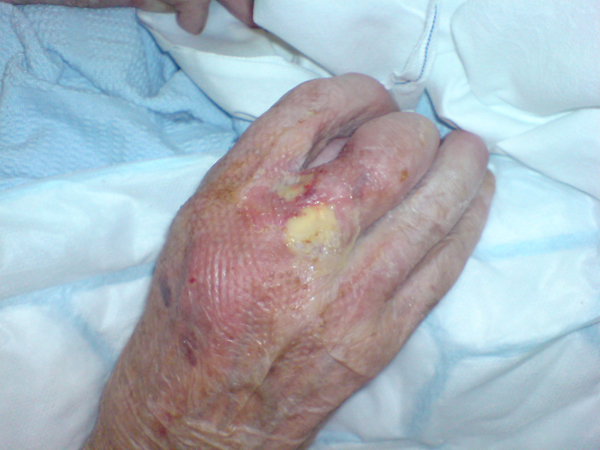
**Discharging lesion on hand**.

He returned four weeks later, complaining of fever, left knee pain and a pus-like discharge from his hands and feet. Blood and knee-joint fluid cultures were negative. An x-ray of his knees showed features of osteoarthritis. A whole-body bone scan excluded the possibility of bone metastases. A few days later, the patient developed multiple tender red, brownish nodules on his lower limbs and discharging lesions on his hands (Figures 2 and 3). He also had a discharging wound on his right buttock. He was referred to the Department of Dermatology where the nodules were thought to represent paraneoplastic lesions or a skin manifestation of atypical *Mycobacterium* infection. The first skin biopsy showed non-specific inflammation of erythema nodosum.

A second skin biopsy revealed extensive areas of necrotic subcutaneous fat surrounded by florid acute and chronic inflammation consistent with pancreatic panniculitis (Figure 3). The patient declined investigations and treatment of the pancreatic mass. Management was primarily supportive and his condition deteriorated rapidly. He was eventually referred to the palliative care team for pain and symptom control. The patient died 5 months after his initial presentation to our hospital.

**Figure 4:**
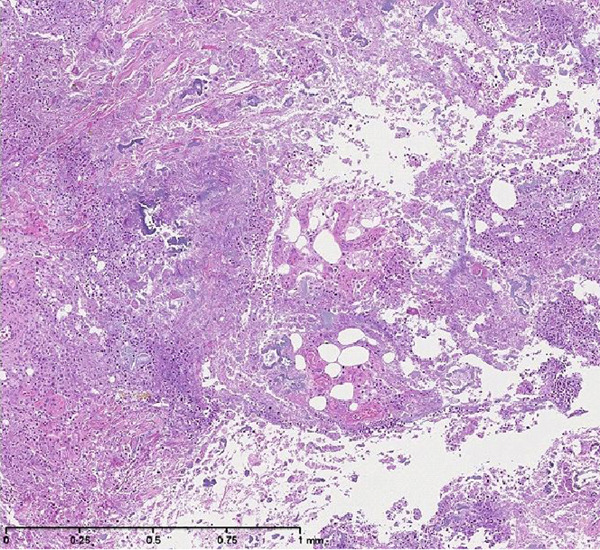
**Histopathological sample showing areas of fat necrosis (haematoxylin stain, ×100)**.

## Discussion

Pancreatic panniculitis affects 2% to 3% of patients with pancreatic disease [2]. This involves fat necrosis in the panniculus and distant foci in pancreatic diseases such as acute or chronic pancreatitis and pancreatic carcinoma. It is less commonly associated with pancreatic pseudocyst, post-traumatic pancreatitis, pancreatic divisum or haemophogocytic syndrome [3]. The pathogenesis is unknown but has been postulated to involve the release of pancreatic enzymes, particularly amylase and lipase from the diseased pancreas, causing subcutaneous fat necrosis [4]. Skin histopathology findings of panniculitis are virtually pathognomonic, described as ghost-like anucleated cells with shadowy walls [5]. Panniculitis manifests as tender erythematous nodules on the lower extremities but can also occur on the arms, trunk or back. These lesions may ulcerate and extrude an oily discharge [6]. Skin lesions are the presenting feature in about 40% of pancreatic panniculitis and in some cases precede development of pancreatic disease [2]. There may be joint manifestations of arthritis or arthralgia [7]. Not much is known of the underlying pathogenesis of arthropathy in pancreatic panniculitis. Some studies postulate that increased levels of lipolytic enzymes of pancreatic origin, once they freely circulate in the tissues, are cytotoxic for many cells including leukocytes and therefore perpetuate inflammation. Synovial fluid of patients with pancreatic arthritis syndrome has been shown to contain elevated concentrations of these lipolytic enzymes [8,9].

Several cases of a triad of panniculitis, pancreatitis and polyarthritis have been reported. Development of pancreatitis in these cases was related to high levels of alcohol intake [10]-[12]. Treatment is largely supportive but, where possible, should involve treatment of the underlying cause. Pancreatic panniculitis has a high mortality rate unless the underlying pancreatic abnormality is reversed [8]. Several case studies have shown resolution of panniculitis in patients after surgical intervention or medical management of their underlying pancreatic disease [3,13]. Prognosis is worse in cases of pancreatic panniculitis associated with pancreatic tumour [14].

In our patient, the clinical finding of panniculitis, pancreatic mass on CT scan, elevated pancreatic enzymes and histological findings of pancreatic panniculitis strengthened the likelihood of a pancreatic tumour in the absence of histopathological and post-mortem biopsies of the pancreatic mass.

## Conclusion

As patients may present to practitioners of various medical specialities with differing symptoms or signs, awareness of the relationship between panniculitis lesions, pancreatic disease and arthritis or arthralgia may prevent a delay of prompt diagnosis and management.

## Consent

As the patient died before the write-up of his case, written informed consent was obtained from the patient's next-of-kin for the publication of this case report and any accompanying images. A copy of the written consent is available for review by the Editor-in-Chief of this journal.

## Competing interests

The author declares that she has no competing interests.
